# Subclinical and overt hypothyroidism is associated with reduced glomerular filtration rate and proteinuria: a large cross-sectional population study

**DOI:** 10.1038/s41598-018-19693-4

**Published:** 2018-02-01

**Authors:** Yi-Cheng Chang, Chia Hsuin Chang, Yi-Chun Yeh, Lee-Ming Chuang, Yu-Kang Tu

**Affiliations:** 1 Graduate Institute of Medical Genomics and Proteomics, National Taiwan University, Taipei, Taiwan; 2 Department of Internal Medicine, National Taiwan University Hospital, Taipei, Taiwan; 3 Department of Medicine, College of Medicine, National Taiwan University, Taipei, Taiwan; 4 Institue of Biomedical Science, Academia Sinica, Taipei, Taiwan; 5 Institute of Epidemiology and Preventive Medicine, College of Public Health, National Taiwan University, Taipei, Taiwan

## Abstract

Subclinical hypothyroidism has been associated with dyslipidemia, hypertension, and increased risk of coronary artery disease. However, limited is known for its effect on renal function. Here we aimed to investigate whether subclinical hypothyroidism is associated with reduced estimated glomerular filtration rate (eGFR) and proteinuria in the general population. A cross-sectional cohort of 74,356 adults aged ≥20 year participating in voluntary health examinations without previous thyroid diseases were recruited in Taiwan. The mean eGFR of persons with euthyroidism, subclinical, and overt hypothyroidism are 87.99, 83.46, and 72.22 mL/min/1.73 m2, respectively (P-for- trend < 0.001). The proportion of proteinuria in persons with euthyroidism, subclinical and overt hypothyroidism is 1.29%, 2.2%, and 2.97%, respectively (P-for-trend: 0.001). The odds ratio of CKD for subclinical, clinical, and all hypothyroidism is 2.04 (95% confidence interval (CI): 1.67-2.50) and 7.61 (95% CI: 4.92-11.77), and 2.41 (95% CI: 2.01–2.89), respectively as compared to euthyroidism. These odd ratios remained significant after further adjustments. The odds ratios for proteinuria is 2.04 (95% CI: 1.67–2.50), 7.61 (95% CI: 4.92–11.77), and 2.41 (95% CI: 2.01–2.89) for subclinical, clinical, and total hypothyroidism, respectively, although the odds ratios were attenuated after further adjustment. Our results suggest subclinical hypothyroidism is a novel risk factor of reduced renal function but not proteinuria.

## Introduction

The concept of mild or subclinical hypothyroidism has emerged in recent decades with the advent of advanced technique to detect subtle changes in thyroid function. It is estimated that 4 to 20 percent of general population have subclinical hypothyroidism, which is defined by elevated thyroid-stimulating hormone (TSH) levels and normal serum thyroxine levels^1–4^. Several studies showed that subclinical hypothyroidism is associated with dyslipidemia, hypertension, accelerated atherosclerosis, and coronary artery disease^5–7^. Even slight elevation of TSH levels have been shown to be associated with accelerated atherosclerosis^5–7^.

Experimental studies showed that deficiency of thyroid hormone causes hypotension^8^, increases vascular resistance^9^, and reduces renal sodium reabsorption^10–12^, which lead to volume contraction and reduced renal blood flow^13^. Sporadic cases of reversible reduced glomerular filtration rate secondary to overt hypothyroidism have been reported^14–17^. Furthermore, hypothyroidism has been shown to cause edema in humans owing to increased capillary permeability to protein, which is reversed by thyroid hormone treatment^18^. These data suggest that hypothyroidism may reduce glomerular infiltration rate and promote urine protein loss.

There are, however, a limited number of studies investigating the impact of subclinical or clinical hypothyroidism on estimated glomerular filtration rate (eGFR) and proteinuria in the general population. Here we investigate the association of subclinical and clinical hypothyroidism with eGFR and proteinuria in a large Taiwanese cohort.

## Results

**Figure 1:**
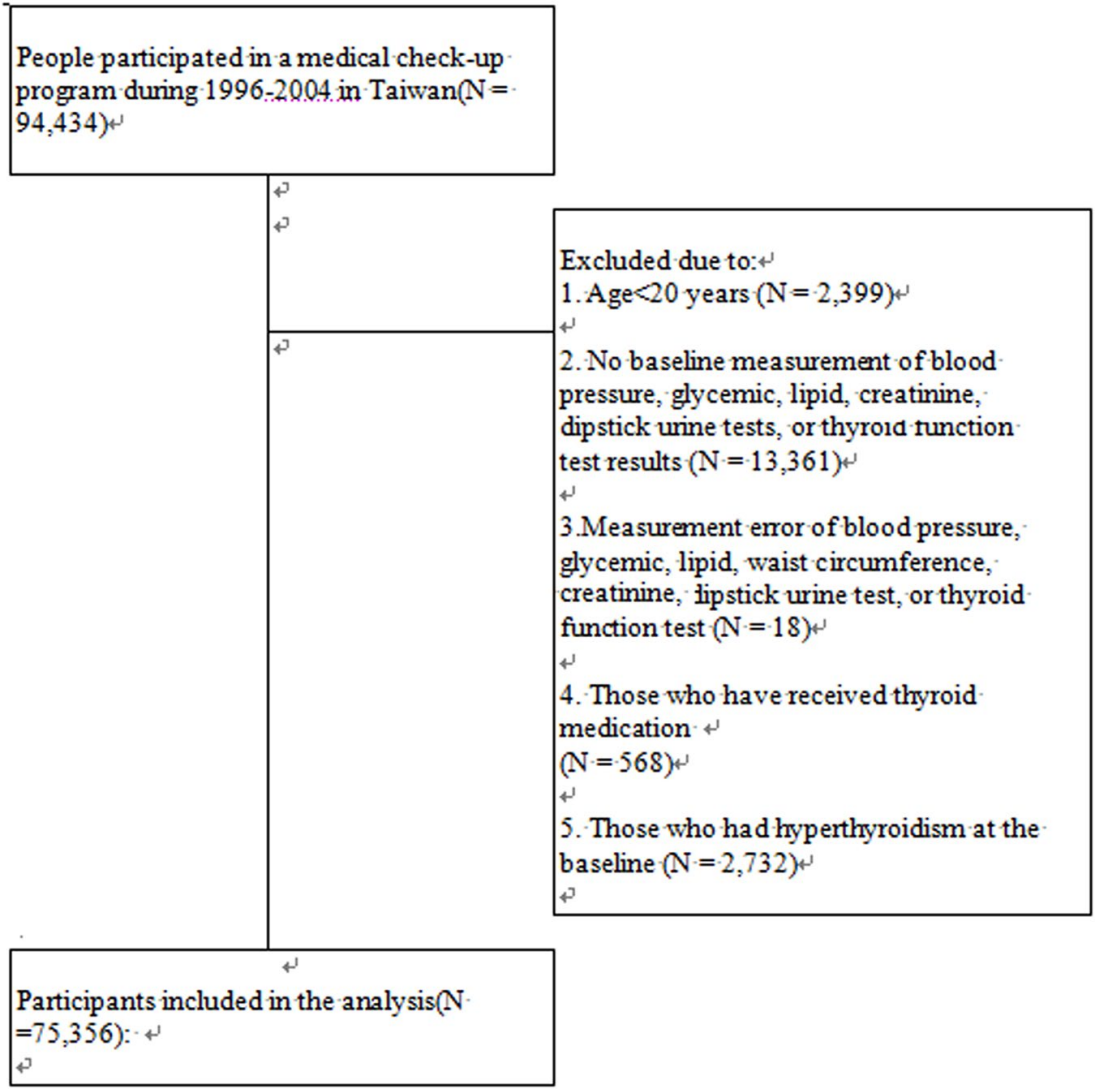
Study flow.

After excluding subjects who did not meet our study criteria, a total of 74,356 participants were included in the analysis (Fig. 1). Among them, 1,240 (1.7%) already had thyroid hypofunction, 3,613(4.9%) already had eGFR < 60 mL/min/1.73 m^2^, and 973 (1.3%) had proteinuria grade ≥ 1+.

Table 1 summarizes the characteristics of participants with normal thyroid function, subclinical, and overt hypothyroidism. When compared to participants with euthyroid state, those with subclinical and overt hypothyroidism were older, had lower proportion of men, and had significantly higher median BMI, blood pressure, fasting glucose, serum triglycerides, total cholesterol, and uric acid levels. They were also more likely to have a lower income and lower educational level. The mean eGFR was 87.99, 83.46, and 72.22 mL/min/1.73 m^2^ for euthyroid, subclinical, and overt hypothyroid participants (P for trend < 0.001). Meanwhile, the proportion of proteinuria grade > 1+ in persons with euthyroidism, subclinical and overt hypothyroidism is 1.29%, 2.2%, and 2.97%, respectively (P for trend = 0.001).

**Table 1:** Characteristics of study participants with euthyroidism, subclinical hypothyroidism, and overt hypothyroidism (N = 74,356).

Variable	All Participants (N = 74,356)						
	Euthyroidism	Subclinical hypothyroidism	Overt hypothyroidism	Total hypothyroidism	P value Euthyroidism vs. subclinical hypothyroidism	P value Euthyroidism vs. overt hypothyroidism	P for trend (euthyroidism, subclinical, and overt hypothyroidism)
Number	73116	1139	101	1240	**—**	**—**	
Male (number, %)	36672 (50.2)	349 (30.6)	30 (29.7)	379 (30.6)	**<0.001**	**<0.001**	**<0.001**
Age (years)							
Mean (SD)	41.7 (13.4)	46.41 (14.1)	50.71 (13.7)	46.76 (14.1)	**<0.001**	**<0.001**	**<0.001**
Age ≥ 40 (number, %)	35142 (48.1)	743 (65.2)	76 (75.3)	819 (66.1)	**<0.001**	**<0.001**	**<0.001**
BMI (kg/m2)							
Mean (SD)	23.10 (3.44)	23.61 (3.66)	24.14 (3.23)	23.65 (3.63)	**<0.001**	**0.002**	**0.002**
Systolic blood pressure (mmHg)							
Mean (SD)	119.57 (19.00)	123.94 (21.60)	125.71 (25.85)	124.09 (21.97)	**<0.001**	**0.019**	**0.001**
Diastolic blood pressure (mmHg)							
Mean (SD)	72.86 (11.30)	74.78 (12.05)	76.03 (13.43)	74.88 (12.17)	**<0.001**	**0.020**	**0.005**
Mean blood pressure (mmHg)							
Mean (SD)	96.20 (14.03)	99.32 (15.75)	100.60 (17.91)	99.43 (15.93)	**<0.001**	**0.016**	**0.002**
Fasting glucose (mg/dL)							
Mean (SD)	98.26 (21.23)	101.43 (30.17)	100.92 (27.98)	101.39 (29.99)	**<0.001**	0.341	0.211
Triglycerides (mg/dL)							
Mean (SD)	114.73 (77.89)	126.73 (80.19)	149.86 (105.82)	128.62 (82.77)	**<0.001**	**0.001**	**<0.001**
Total cholesterol (mg/dL)							
Mean (SD)	194.93 (36.85)	201.91 (39.43)	246.05 (69.25)	205.50 (44.28)	**<0.001**	**<0.001**	**<0.001**
Uric acid (mg/dL)							
Mean (SD)	6.06 (1.62)	5.86 (1.62)	6.03 (1.56)	5.87 (1.61)	**<0.001**	0.878	0.878
TSH (mU/L)							
Mean (SD)	1.53 (0.78)	7.90 (5.24)	55.21 (61.75)	11.75 (22.37)	**<0.001**	**<0.001**	**<0.001**
T4 (nM)							
Mean (SD)	7.80 (1.47)	7.22 (1.45)	2.80 (1.45)	6.86 (1.89)	**<0.001**	**<0.001**	**<0.001**
eGFR (ml/min)							
Mean (SD)	87.99 (17.16)	83.46 (18.06)	72.22 (18.76)	82.55 (18.37)	**<0.001**	**<0.001**	**<0.001**
Proteinuria (number, %)							
Normal	67916 (92.89)	1042 (91.48)	92 (91.09)	1134 (91.45)	0.066	**0.012**	**0.001**
Trace	4255 (5.82)	72 (6.32)	6 (5.94)	78 (6.29)			
1+	575 (0.79)	14 (1.23)	0	14 (1.13)			
2+	235 (0.32)	6 (0.53)	2 (1.98)	8 (0.65)			
3+	135 (0.18)	5 (0.44)	1 (0.99)	6 (0.48)			
Hypertension (number, %)	10627 (14.53)	236 (20.72)	24 (23.76)	260 (20.97)	**<0.001**	**0.009**	**<0.001**
Total dysglycemia	21520 (29.43)	379 (33.27)	35 (34.65)	414 (33.39)	**0.003**	0.493	**0.001**
Pre-diabetes (number, %)	18200 (24.89)	308 (27.04)	29 (28.71)	337 (27.18)			
Diabetes (number, %)	3320 (4.54)	71 (6.23)	6 (5.94)	77 (6.21)			
Hypercholesterolemia (number, %)	8389 (11.47)	170 (14.93)	46 (45.54)	216 (17.42)	**<0.001**	**<0.001**	**<0.001**
Hypertriglyceridemia (number, %)	15599 (21.33)	297 (26.08)	43 (42.57)	340 (27.42)	**<0.001**	**<0.001**	**<0.001**
Low-HDL cholesterol level (number, %)	28920 (39.55)	490 (43.02)	41 (40.59)	531 (42.82)	**0.018**	0.831	**0.032**
Abdominal adiposity (number, %)							
No	54591 (74.66)	760 (66.73)	61 (60.40)	821 (66.21)	**<0.001**	**<0.001**	**<0.001**
Yes	18524 (25.34)	379 (33.27)	40 (39.60)	419 (33.79)			
Missing	1	0	0	0			
Hyperuricemia (number, %)	23328 (31.91)	374 (32.84)	42 (41.58)	416 (33.55)	0.504	0.037	0.101
Low education level (number, %)							
No	50665 (71.16)	632 (57.04)	44 (45.36)	676 (56.10)	**<0.001**	**<0.001**	**<0.001**
Yes	20533 (28.84)	476 (42.96)	53 (54.64)	529 (43.90)			
Missing	1918	31	4	35			
Income level (number, %)							
Low	14607 (41.23)	310 (56.99)	27 (57.45)	337 (57.02)	**<0.001**	0.078	**<0.001**
Middle	12704 (35.86)	139 (25.55)	12 (25.53)	151 (25.55)			
High	8116 (22.91)	95 (17.46)	8 (17.02)	103 (17.43)			
Missing	37689	595	54	649			
Cigarette smoking (number, %)							
No	46893 (70.81)	835 (81.30)	70 (76.09)	905 (80.88)	**<0.001**	0.265	**<0.001**
Yes	19335 (29.19)	192 (18.70)	22 (23.91)	214 (19.12)			
Missing	6888	112	9	121			
Regular alcohol consumption (number, %)							
No	64036 (95.39)	978 (96.74)	89 (98.89)	1067 (96.91)	**0.042**	0.113	0.011
Yes	3095 (4.61)	33 (3.26)	1 (1.11)	34 (3.09)			
Missing	5985	128	11	139			
Physical inactivity (number, %)							
No	36759 (52.76)	565 (51.83)	43 (46.24)	608 (51.39)	0.545	0.208	0.248
Yes	32916 (47.24)	525 (48.17)	50 (53.76)	575 (48.61)			
Missing	3441	49	8	57			

In Tables 2 and 3, we summarized the characteristics of participants with different eGFR and proteinuria status. There is a significantly increased proportion of subclinical, overt, and total hypothyroidism with decreasing eGFR level (P value for trends <0.001, 0.001, and <0.001, respectively) and an increased severity of proteinuria (P value for trend =0.0071, 0.0703, and 0.0020, respectively).

**Table 2:** Characteristics among study participants with different eGFR levels (N = 74,356).

Variable	Number of participants for evaluation	Kidney function impairment								
		eGFR ≥ 90		eGFR 60–89		eGFR 30–59		eGFR < 30		P for trend
		n	(%)	n	(%)	n	(%)	n	(%)	
Total	74356	32878	(100.0)	37865	(100.0)	3487	(100.0)	126	(100.0)	
Age ≥ 40	74356	8893	(27.0)	23583	(62.3)	3372	(96.7)	113	(89.7)	**<0.001**
Male	74356	13089	(39.8)	21764	(57.5)	2130	(61.1)	68	(54.0)	**<0.001**
Lower educational level	72403	5530	(17.2)	13330	(36.3)	2121	(63.8)	81	(67.5)	**<0.001**
Low income level	36018	7163	(41.3)	6893	(39.8)	857	(64.9)	31	(77.5)	**<0.001**
BMI ≥ 27	74350	2992	(9.1)	5462	(14.4)	701	(20.1)	18	(14.3)	**<0.001**
Hypertension	74356	2423	(7.4)	6841	(18.1)	1543	(44.3)	80	(63.5)	**<0.001**
Total dysglycemia	74356	7142	(21.7)	12995	(34.3)	1731	(49.6)	66	(52.4)	**<0.001**
Diabetes	74356	867	(3.3)	2018	(7.5)	492	(21.9)	20	(25.0)	**<0.001**
Pre-diabetes	74356	6275	(19.6)	10977	(30.6)	1239	(41.4)	46	(43.4)	**<0.001**
Hypercholesterolemia	74356	2531	(7.7)	5260	(13.9)	777	(22.3)	37	(29.4)	**<0.001**
Hypertriglyceridemia	74356	4860	(14.8)	9626	(25.4)	1392	(39.9)	61	(48.4)	**<0.001**
Low-HDL cholesterol level	74356	12567	(38.2)	15224	(40.2)	1590	(45.6)	70	(55.6)	**<0.001**
Abdominal adiposity	74355	5993	(18.2)	11165	(29.5)	1720	(49.3)	65	(51.6)	**<0.001**
Hyperuricemia	74356	7595	(23.1)	14089	(37.2)	1978	(56.7)	82	(65.1)	**<0.001**
Total hypothyroidism	74356	426	(1.3)	681	(1.8)	128	(3.7)	5	(4.0)	**<0.001**
Overt hypothyroidism	74356	17	(0.1)	56	(0.2)	26	(0.8)	2	(1.6)	**<0.001**
Subclinical hypothyroidism	74356	409	(1.2)	625	(1.7)	102	(2.9)	3	(2.4)	**<0.001**
Physical inactivity	70872	16945	(53.5)	15389	(43.0)	1118	(34.4)	40	(35.4)	**<0.001**
Cigarette smoking	67372	7117	(23.7)	11304	(33.1)	1097	(35.8)	37	(33.3)	**<0.001**
Alcohol consumption	68253	1225	(4.0)	1810	(5.2)	90	(2.9)	4	(3.7)	**<0.001**

**Table 3:** Characteristics among study participants with different severity of proteinuria (N = 74,356).

Variable	Number of participants for evaluation	Proteinuria										
		Normal		+−		1+		2+		3+		P for trend
		n	(%)	n	(%)	n	(%)	n	(%)	n	(%)	
Total	74356	69050	(100.0)	4333	(100.0)	589	(100.0)	243	(100.0)	141	(100.0)	—
Age ≥ 40	74356	32985	(47.8)	2256	(52.1)	439	(74.5)	175	(72.0)	106	(75.2)	**<0.001**
Male	74356	34250	(49.6)	2298	(53.0)	301	(51.1)	126	(51.9)	76	(53.9)	**<0.001**
Lower educational level	72403	19161	(28.5)	1406	(33.5)	291	(51.4)	125	(53.4)	79	(58.1)	**<0.001**
Low income level	36018	13773	(40.9)	944	(48.3)	136	(53.1)	48	(53.3)	43	(68.3)	**<0.001**
BMI ≥ 27	74350	8162	(11.8)	727	(16.8)	171	(29.0)	68	(28.0)	45	(31.9)	**<0.001**
Hypertension	74356	9388	(13.6)	993	(22.9)	292	(49.6)	130	(53.5)	84	(59.6)	**<0.001**
Total dysglycemia	74356	19741	(28.6)	1635	(37.7)	332	(56.4)	132	(54.3)	94	(66.7)	**<0.001**
Diabetes	74356	2629	(5.1)	486	(15.3)	164	(39.0)	63	(36.2)	55	(53.9)	**<0.001**
Pre-diabetes	74356	17112	(25.8)	1149	(29.9)	168	(39.5)	69	(38.3)	39	(45.3)	**<0.001**
Hypercholesterolemia	74356	7801	(11.3)	551	(12.7)	127	(21.6)	70	(28.8)	56	(39.7)	**<0.001**
Hypertriglyceridemia	74356	14271	(20.7)	1197	(27.6)	268	(45.5)	121	(49.8)	82	(58.2)	**<0.001**
Low-HDL cholesterol level	74356	26944	(39.0)	2039	(47.1)	287	(48.7)	111	(45.7)	70	(49.6)	**<0.001**
Abdominal adiposity	74355	17084	(24.7)	1371	(31.6)	302	(51.3)	113	(46.5)	73	(51.8)	**<0.001**
Hyperuricemia	74356	21695	(31.4)	1525	(35.2)	310	(52.6)	131	(53.9)	83	(58.9)	**<0.001**
Total hypothyroidism	74356	1134	(1.6)	78	(1.8)	14	(2.4)	8	(3.3)	6	(4.3)	**0.0020**
Overt hypothyroidism	74356	92	(0.1)	6	(0.1)	0	(0)	2	(0.8)	1	(0.7)	0.0703
Subclinical hypothyroidism	74356	1042	(1.5)	72	(1.7)	14	(2.4)	6	(2.5)	5	(3.6)	**0.0071**
Physical inactivity	70872	31067	(47.2)	2027	(49.6)	246	(44.9)	94	(41.6)	58	(44.6)	0.7231
Cigarette smoking	67372	17847	(28.5)	1434	(36.9)	153	(29.8)	75	(36.1)	46	(36.2)	**<0.001**
Alcohol consumption	68253	2800	(4.4)	275	(7.0)	33	(6.3)	14	(6.5)	7	(5.6)	**<0.001**

In the univariate analysis, subclinical, overt, and total hypothyroidism was associated with impaired renal function defined by eGFR < 60 mL/min/1.73 m^2^ and the crude ORs (95% CI) were 2.03 (1.66–2.49), 7.68 (4.96–11.89), and 2.40 (2.00–2.89) respectively, and for proteinuria grade ≥ 1+, the crude ORs (95% CI) were 1.71 (1.15–2.56), 2.35 (0.74–7.40), and 1.76 (1.21–2.58), respectively (Table 4). After controlling for the potential risk factors, participants with total hypothyroidism still had a significant 92% increase in the risk of eGFR < 60 mL/min/1.73 m^2^ (the adjusted OR = 1.92; 95% CI: 1.35–2.71), but the association with proteinuria was attenuated (adjusted OR = 1.32; 95% CI 0.73–2.42) after adjustment of potential risk factors (Table 4).

**Table 4:** Association between hypothyroidism and renal function impairment/proteinuria (N = 74,356).

Model	eGFR < 60			Proteinuria ≥ 1+		
	Subclinical hypothyroidism	Overt hypothyroidism	Total hypothyroidism	Subclinical hypothyroidism	Overt hypothyroidism	Total hypothyroidism
Crude OR (euthyroidism as reference)	2.03 (1.66–2.49)	7.68 (4.96–11.89)	2.40 (2.00–2.89)	1.71 (1.15–2.56)	2.35 (0.74–7.40)	1.76 (1.21–2.58)
Model 1: age and sex	1.53 (1.21–1.93)	5.67 (3.38–9.53)	1.83 (1.48–2.26)	1.40 (0.94–2.10)	1.62 (0.51–5.15)	1.42 (0.97–2.09)
Model 2: Model 1+ mean blood pressure, fasting glucose, BMI, total cholesterol, triglycerides, uric acids	1.51 (1.19–1.93)	4.82 (2.76–8.40)	1.78 (1.43–2.21)	1.14 (0.74–1.75)	1.10 (0.32–3.75)	1.13 (0.75–1.70)
Model 3: Model 2+ physical inactivity, cigarette smoking, alcohol consumption	1.48 (1.13–1.93)	3.56 (1.90–6.66)	1.67 (1.30–2.13)	1.19 (0.75–1.91)	0.78 (0.17–3.49)	1.14 (0.73–1.80)
Model 4: model 3+ low income level, low education level	1.76 (1.21–2.58)	3.13 (1.30–7.53)	1.92 (1.35–2.72)	1.25 (0.65–2.40)	1.93 (0.44–8.57)	1.32 (0.73–2.42)
Model 6: Model 4+ proteinuria	1.74 (1.18–2.56)	3.16 (1.30–7.67)	1.89 (1.33–2.71)	—	—	—
Model 6: Model 4+ eGFR	—	—	—	1.03 (0.53–2.02)	1.37 (0.30–6.20)	1.08 (0.58–2.00)

To explore the interaction between proteinuria and eGFR, the association of hypothyroidism and eGFR < 60 mL/min/1.73 m^2^ was further adjusted by proteinuria (Table 4, model 6) and the association remain similar and significant. Conversely, the association between hypothyroidism and proteinuria were also further adjusted by eGFR. This association was significantly diminished (Table 4, model 6).

In sensitivity analysis excluding participants who reported to receive anti-hypertensives, anti-diabetics, lipid-lowering agents, uricosuric agents, or thyroid medication, the association between hypothyroidism and the risk of eGFR < 60 mL/min/1.73 m^2^ were more pronounced (Supplementary Tables 2 & 3).

In the stratified analyses aimed to evaluate whether risks were modified by baseline characteristics, hypothyroidism was consistently and significantly associated with an increased risk of eGFR < 60 mL/min/1.73 m^2^ across all age and sex groups (Supplementary Table 4). Female subjects and younger subjects (age 20–49 years-old) seem to have higher ORs of decreased eGFR than males or older subjects (age ≥ 50 years) (Supplementary Table 4). Hypothyroid participants without dysglycemia had a slightly higher OR of decreased eGFR than those with dysglycemia (Supplementary Table 4).

## Discussion

In this large cross-sectional study involving 74,356 participants recruited from a voluntary health examination program, we found a clear dose-dependent association between hypothyroid status and eGFR and proteinuria. The risk of CKD, defined as eGFR less than 60 mL/min/1.73 m^2^, increased by 2.03 and 7.68-fold for subclinical and overt hypothyroidism as compared to euthyroidism, which remained significant after adjustment for other potential risk factors of CKD. The risk of proteinuria increases by 1.71 and 2.35-fold for subclinical and overt hypothyroidism, but it was attenuated by further adjustment. Our study demonstrates the association of subclinical and overt hypothyroidism with reduced renal function and, to a lesser extent, proteinuria in the general population. To our knowledge, this is the largest study to explore the association between hypothyroidism and eGFR/proteinuria in the general population.

Our results are consistent with previous studies showing that the prevalence of subclinical or clinical hypothyroidism increased as eGFR decreased in the general population^19–21^. In the National Center for Health Statistics third national survey (NHANES III) involving 14, 623 adults, the prevalence of hypothyroidism, including both subclinical and clinical hypothyroidism are 5.4%, 10.9%, 20.4%, 23.0%, and 23.1% in subjects with eGFR >90, 60–89, 45–59, 30–44, and <30 mL/min/1.73 cm^2^, respectively^19^. In the Nord-Tronderlag Health Study (HUNT) study involving 24,980 adults in Norway, the mean eGFR is 83.0, 81.6, and 80.3 mL/min/1.73 cm^2^ in euthyroid subjects with TSH levels of the lower, middle, and higher thirds^20^. The eGFR decreased further to 79.3 and 76.5 mL/min/1.73 cm^2^ in subjects with subclinical and overt hypothyroidism^20^. In another Italian study recruiting 3,089 participants, the prevalence of subclinical hypothyroidism increased from 7% in participants with eGFR > 90 mL/min/1.73 cm^2^ to 17.9% in participants with eGFR < 60 mL/min/1.73 cm^2^ ^21^. These data, together with our present study, support the association of subclinical and clinical hypothyroidism with reduced eGFR in the general population. Collectively, these data suggest subclinical hypothyroidism as a novel risk factor of reduced renal function.

Because of the cross-sectional design of these studies, the causal relationship between hypothyroidism and reduced eGFR remains debatable. In uremic patients, low free T3 (low T3 syndrome) resulting form impaired peripheral synthesis of T3 from T4 is the most frequently observed hormonal change^22–24^. However, these hormonal changes were observed in patients with advanced CKD. In our analysis, the prevalence of subclinical and clinical hypothyroidism increase steadily from very-early-stage CKD to advanced CKD. It seems unlikely that only mild decrement in eGFR would affect thyroid function.

Conversely, several observational studies support the notion that hypothyroidism is the culprit of reduced eGFR. Sporadic cases of reversible renal impairment secondary to hypothyroidism have been repeatedly reported^14–17^. In addition, several case series reported improved renal function by thyroxine treatment in patients with overt hypothyroidism^25–27^. These observations indicate that hypothyroidism reduces eGFR, which could be reversed by thyroxine treatment. In a retrospective study, Shin, *et al*. further demonstrated that thyroxine treatment in patients with subclinical hypothyroidism and CKD significantly lowered the rate of decline in eGFR than those not receiving treatment^28,29^. The protective effect remained significant even after adjustment of other risk factors. Another prospective follow-up study involving 41,454 Taiwanese elderly adults with age >65 year, those with subclinical hypothyroidism have increased HR (1.15; 95% C.I.: 1.05–1.26) of developing CKD and those with over hypothyroidism have even higher increased HR (1.27; 95% C.I.:1.06–1.16) of developing CKD^30^. In consistent with our stratified analyses, female hypothyroid subjects are more prone to develop CKD in this study (Supplementary Table 4)^9,30–32^. This study, together with our results suggests thyroid hormone might improve renal function in patients with subclinical hypothyroidism.

Several mechanisms by which hypothyroidism dampens glomerular filtration rate were proposed. Mice deficient for thyroid hormone receptor developed hypotension and bradycardia, thereby reducing cardiac output and renal perfusion^8^. Hypothyroid rats also have a higher renal excretion of sodium^10,11^. Administration of thyroid hormone to hypothyroid rats restored proximal tubule sodium reabsorption due to an increase in Na+/H+ antiporter activity^12^, which lead to volume expansion^13^. In addition, thyroid hormone has been shown to relax arteries and reduce arterial resistance^9^. These actions of thyroid hormone results in increased glomerular filtration rate^13^.

Despite previous studies consistently demonstrating the link between hypothyroidism and decreased eGFR, none of them explore the role of hypothyroidism in proteinuria. Our study is the only one to investigate the role of hypothyroidism in the proteinuria. Our study showed the severity of proteinuria increase progressively from euthyroidism, subclinical to overt hypothyroidism. The risk of proteinuria is increased by 1.71-fold in patients with subclinical hypothyroidism and 2.35-fold in patients with overt hypothyroidism. The same albeit less significant trend was observed after further adjustment for other risk factors. The less significant association may result from increased missing values when multiple covariates are incorporated in regression models. To our knowledge, our study is the first study to examine the association between hypothyroidism and proteinuria in the general population.

The causal relationship between hypothyroidism and proteinuria is also uncertain. In patients with nephrotic syndrome, the heavy urinary loss of thyroid hormone-binding proteins, including thyroxine binding globulin, thransthyretin, and albumin, results in a reduction in total T4^31^. However, the thyroid gland is able to compensate the loss so that most patients remained in euthyroid state since serum free T4 or free T3 levels remain normal^31^. In a study involving 159 patients with nephrotic syndrome and 900 controls, nephrotic syndrome patients have slightly elevated TSH in normal range (1.81 v.s. 1.34 mIU/L, P < 0.001) and similar free T4 (13.1 v.s, 13.1 pmol/L) levels as compared to controls^32^. In our analysis, the prevalence of subclinical or clinical hypothyroidism was increased steadily from even trace to heavy proteinuria. The possibility of trace or mild proteinuria cause hypothyroidism seems unlikely.

Conversely, hypothyroidism has been found to be associated with a variety of glomerulopathy including membranous glomerulopathy, minimal change nephritic syndrome and membranoproliferative glomerulonephritis, and thyroxine treatment alleviated the urine protein loss^33–37^. Even in subclinical hypothyroidism patients, thyroxine treatment has been shown to reduce edema, capillary permeability of albumin, and plasma colloid osmotic pressure, suggesting deficiency of thyroid hormone may increase vascular permeability to protein^18^. Alternatively, a common autoimmune background may explain the link between hypothyroidism and glomerulopathy because autoimmune thyroiditis is the most frequent cause of hypothyroidism and the glomerulopathy observed in hypothyroid patients are often caused by immune complex deposition.

Our present study has several strengths. This is the largest study to investigate the association between subclinical/clinical hypothyroidism and eGFR and is the first study to examine the association between subclinical/clinical hypothyroidism and proteinuria in the general population. The clinical examination and lab test are performed as screening assessments rather than for a clinical indication. The comprehensive coverage of clinical examinations and lab test enable adjustments for multiple confounding factors.

Our study has several limitations. First, this is a cross-sectional study. Therefore the causal relationship cannot be established. Although we have comprehensively adjusted for possible confounding factors, a longitudinal follow-up study is required to address this issue. Second, we did not measure thyroid autoantibody or other autoimmune antibodies. Therefore, the possibility of a common underlying autoimmune process linking both hypothyroidism and proteinuria cannot be excluded. Third, the participants are recruited from voluntary health examinations, which may not be a representative sample. Therefore, the estimation of prevalence may not be accurate. Fourthly, the urine dipstick tests are only semi-quantitative measure of urine protein loss. Information about 24-hr urine protein loss is not available in our study. Lastly, missing values in various lab tests reduce the sample size in multivariate regression model and the statistical power, which may render the results of multivariate regression less significant.

In conclusion, we found subclinical and clinical hypothyroidism is independently associated reduced eGFR in a dose-dependent manner. A similar but less significant association was also found with proteinuria. Together with previous studies, these results suggest subclincal hypothyroidism is a novel risk factor of impaired renal function, which is reversible and often neglected.

## Methods

### Data source and study population

In this study, we used data of 94,434 individuals who participated in a voluntary comprehensive medical screening program offered by a private organization (MJ Health Screening Center in Taiwan) between 1996 and 2006^38,39^. Written informed consent for using the screening results for academic research and online open publication were obtained from each participant, and this research project for the uses of those datasets was approved by the Research Ethics Committee in Leeds Institute of Genetics, Health and Therapeutics at the University of Leeds, Leeds, UK. All methods employed in this study were in accordance with the applicable guidelines and regulations. All personal information was kept confidential as required. Information related to personal identification was delinked from the data and participants remained anonymous throughout the analysis. Participants were excluded if they 1) Age < 20 years; 2) did not have complete baseline measurements of blood pressure, glycemic, lipid, creatinine, dipstick urine test, or thyroid function test results; 3) had implausibly large or small values due to measurement error or typing error of blood pressure, glycemic, lipid, waist circumference, creatinine, dipstick urine test, or thyroid function test; 4) reported the use of thyroid medications; and 5) had hyperthyroidism at baseline (Fig. 1).

### Data collection

In addition to a self-administered questionnaire for education level, lifestyle factors, and past medical history, each participant undertook a standard set of medical history, physical examinations, blood, and urine tests. Overnight fasting blood and first morning voided urine were collected and analyzed.

### Definition of hypothyroidism and other covariates

Participants were classified into three groups according to their thyroid function test results: subclinical hypothyroidism (4.5 < T4 < 12 μg/dL and TSH > 5 mIU/L), overt hypothyroidism (T4 < 4.5 μg/dL and TSH > 5 mIU/L), and euthyroidism (4.5 < T4 < 12 μg/dL and 0.47 mIU/L < TSH < 5 mIU/L).Estimated glomerular filtration rate (eGFR) was calculated by the CKD-EPI Study equation^40^. Dysglycemia included diabetes and pre-diabetes. Diabetes was defined as fasting plasma glucose ≥126 mg/dL, self-reported history of diabetes, or use of any anti-diabetes medication. Pre-diabetes was defined as fasting glucose ≥100 mg/dL but <126 mg/dL, no self-reported history of diabetes, nor user of anti-diabetic drugs. Hyperuricemia was defined as a serum uric acid level of more than 7.2 mg/dl in men or 6.0 mg/dl in women. Hypertension was defined by SBP ≥ 140 mmHg, DBP ≥ 90 mmHg, self-reported history of hypertension, or user of antihypertensive drugs. Hypercholesterolemia was defined by total cholesterol ≥ 240 mg/dL or receiving drug therapy for hypercholesterolemia. Hypertriglyceridemia was defined by serum triglycerides ≥ 150 mg/dL or receiving lipid lowering therapy for hypertriglyceridemia. Low HDL-cholesterol was defined by <40 mg/dL in men or < 50 mg/dL in women. Large waist circumference was defined by ≥90 cm (35 inch) in men or ≥80 cm (32 inch) in women. Participants, whose highest education was junior high school or lower, were considered to be of low educational level, while those, who completed senior high school or higher education, were considered to be of high educational level. Income levels were divided into three categories based on an annual income: low (less than 400,000 NT dollars), middle (400,000 to 800,000 NT dollars) and high (more than 800,000 NT dollars). Regular alcohol consumption was defined as having 3 or more drinks per week and more than 2 cups per drink. Reporting no regular exercise was viewed as physical inactivity.

### Statistical analyses

Characteristics of participants with normal thyroid function, subclinical hypothyroidism, and overt hypothyroidism were pairwise compared using t test for continuous variables and chi-square test for categorical variables. A linear trend test was used to examine whether there was an increasing proportion or value of participants with hypothyroidism and other metabolic risk factors with increasing level of renal function impairment and proteinuria. Separate logistic regression models were used to calculate the crude odds ratios (ORs) and 95% confidence intervals (CIs) of eGFR < 60 mL/min/1.73 m^2^ or proteinuria grade ≥ 1+ for participants with subclinical and overt hypothyroidism relative to those with normal thyroid function. Multivariable logistic regression analyses were sequentially conducted to control for additional potential risk factors, including 1) age and sex; 2) mean blood pressure, fasting glucose, BMI, total cholesterol, triglycerides, uric acids; 3) physical inactivity, cigarette smoking, alcohol consumption; and 4) low income level, and low education level.

Age and blood pressure are well-known risk factors for impaired renal function, and the relation between these two factors and eGFR/proteinuria may not be linear. Meanwhile, several published studies reported that certain commonly used medications for diabetes and hypertension may influence TSH level. Therefore, in the subsequent sensitivity analyses, we investigated whether the association between overt hypothyroidism, subclinical hypothyroidism, eGFR < 60 mL/min/1.73 m^2^ and proteinuria ≥1+ would change when 1) spline logistic regression was used to control for age and mean blood pressure (Supplementary Table 1); 2) participants who reported to receive anti-hypertensives, anti-diabetics, lipid-lowering agents, uricosuric agents, or thyroid medications were excluded (Supplementary Table 2); and 3) spine logistic regression was used to control for age and mean blood pressure and participants who reported to receive the above medications were excluded (Supplementary Table 3). Furthermore, stratified analyses were performed to evaluate whether the associations between hypothyroidism, reduced eGFR and proteinuria were moderated by baseline characteristics. Participants were stratified according to 1) sex (men, women); 2) age (20–49 years, ≥50 years); and 3) whether the participants had dysglycemia (diabetes and pre-diabetes) or euglycemia (Supplementary Table 4). The statistical significance level was set at 5%. All statistical analyses were performed with SAS 9.2 (SAS Institute, Cary, NC).

### Data availability

The datasets generated during and/or analyzed during the current study are available from the corresponding author on reasonable request.

## Electronic supplementary material

Supplementary Information
