# Supplementary material for: Subclinical and overt hypothyroidism is associated with reduced glomerular filtration rate and proteinuria: a large cross-sectional population study
Source: Sci Rep. 2018 Feb 1;8:2031. doi: 10.1038/s41598-018-19693-4 (PMC5795015; doi:10.1038/s41598-018-19693-4)
Supplement: Supplementary file 1 — Supplementary Information [file 41598_2018_19693_MOESM1_ESM.doc]

**Supplementary data to**

**Subclinical and overt hypothyroidism is associated with reduced glomerular filtration rate and proteinuria: a large cross-sectional population study**

Chia-Hsuin Chang, Yi-Cheng Chang , Yi-Chun Yeh, Lee-Ming Chuang, Yu-Kang Tu

Supplementary Table 1. Sensitivity analysis: Association between hypothyroidism and renal function impairment/proteinuriausing spline logistic regression controlling for age and mean blood pressure (N = 74,356)

|  | *All age* | | | | | |
| --- | --- | --- | --- | --- | --- | --- |
|  | *eGFR<60* | | | *Proteinuria ≥ 1+* | | |
| *Model* | *Subclinical hypothyroidism* | *Overt hypothyroidism* | *Total hypothyroidism* | *Subclinical hypothyroidism* | *Overt hypothyroidism* | *Total hypothyroidism* |
| Crude OR (euthyroidism as reference) | 2.03 (1.66-2.49) | 7.68 (4.96-11.89) | 2.40 (2.00-2.89) | 1.71 (1.15-2.56) | 2.35 (0.74-7.40) | 1.76 (1.21-2.58) |
| Model 1: age and sex | 1.56 (1.24-1.96) | 4.96 (2.87-8.57) | 1.81 (1.47-2.23) | 1.40 (0.93-2.10) | 1.58 (0.50-5.03) | 1.42 (0.97-2.09) |
| Model 2: Model 1+ mean blood pressure, fasting glucose, BMI, total cholesterol, triglycerides, uric acids | 1.51 (1.18-1.92) | 4.22 (2.38-7.50) | 1.76 (1.41-2.19) | 1.12 (0.72-1.73) | 1.01 (0.29-3.52) | 1.11 (0.73-1.67) |
| Model 3: Model 2+ physical inactivity, cigarette smoking, alcohol consumption | 1.46 (1.12-1.90) | 3.36 (1.80-6.30) | 1.48 (1.16-1.88) | 1.21 (0.75-1.93) | 0.66 (0.14-3.21) | 1.14 (0.73-1.80) |
| Model 4: model 3 +low income level, low education level | 1.73 (1.18-2.53) | 2.96 (1.23-7.15) | 1.87 (1.32-2.65) | 1.29 (0.67-2.49) | 1.78 (0.39-8.04) | 1.35 (0.74-2.47) |

**Supplementary Table 2. Sensitivity analysis: Association between hypothyroidism and renal function impairment/proteinuria after excluding participants** who reported to receive anti-hypertensives, anti-diabetics, lipid-lowering agents, uricosuric agents, or thyroid medications (N = 68,079)

|  | *eGFR<60* | | | *Proteinuria ≥ 1+* | | |
| --- | --- | --- | --- | --- | --- | --- |
| *Model* | *Subclinical hypothyroidism* | *Overt hypothyroidism* | *Total hypothyroidism* | *Subclinical hypothyroidism* | *Overt hypothyroidism* | *Total hypothyroidism* |
| Crude OR (euthyroidism as reference) | 2.29 (1.79-2.93) | 9.63 (5.81-15.97) | 2.76 (2.21-3.44) | 1.67 (0.98-2.86) | 2.89 (0.71-11.80) | 1.77 (1.07-2.92) |
| Model 1: age and sex | 1.70 (1.28-2.26) | 7.58 (4.18-13.74) | 2.10 (1.64-2.71) | 1.49 (0.87-2.55) | 2.33 (0.57-9.52) | 1.56 (0.94-2.58) |
| Model 2: Model 1+ mean blood pressure, fasting glucose, BMI, total cholesterol, triglycerides, uric acids | 1.63 (1.22-2.18) | 7.18 (3.87-13.35) | 2.02 (1.56-2.62) | 1.25 (0.72-2.17) | 1.75 (0.42-7.37) | 1.30 (0.77-2.17) |
| Model 3: Model 2+ physical inactivity, cigarette smoking, alcohol consumption | 1.58 (1.14-2.18) | 5.30 (2.55-11.01) | 1.86 (1.39-2.50) | 1.46 (0.81-2.64) | 1.13 (0.15-8.42) | 1.42 (0.81-2.51) |
| Model 4: model 3 + low income level, low education level | 2.01 (1.27-3.18) | 3.56 (1.21-10.48) | 2.18 (1.42-3.33) | 1.48 (0.63-3.45) | 2.34 (0.29-18.52) | 1.56 (0.71-3.42) |

**Supplementary Table 3. Sensitivity analysis: Association between hypothyroidism and renal function impairment/proteinuria after excluding participants who reported to receive anti-hypertensives, anti-diabetics, lipid-lowering agents, uricosuric agents, or thyroid medications and using spline logistic regression controlling for age and mean blood pressure** (N = 68,079)

|  | *eGFR<60* | | | *Proteinuria ≥ 1+* | | |
| --- | --- | --- | --- | --- | --- | --- |
| *Model* | *Subclinical hypothyroidism* | *Overt hypothyroidism* | *Total hypothyroidism* | *Subclinical hypothyroidism* | *Overt hypothyroidism* | *Total hypothyroidism* |
| Crude OR (euthyroidism as reference) | 2.29 (1.79-2.93) | 9.63 (5.81-15.97) | 2.76 (2.21-3.44) | 1.67 (0.98-2.86) | 2.89 (0.71-11.80) | 1.77 (1.07-2.92) |
| Model 1: age and sex | 1.69 (1.28-2.23) | 7.28 (4.01-13.24) | 2.08 (1.62-2.67) | 1.48 (0.87-2.53) | 2.28 (0.56-9.33) | 1.55 (0.94-2.56) |
| Model 2: Model 1+ mean blood pressure, fasting glucose, BMI, total cholesterol, triglycerides, uric acids | 1.64 (1.23-2.19) | 6.90 (3.71-12.84) | 2.00 (1.54-2.58) | 1.23 (0.70-2.14) | 1.65 (0.39-7.00) | 1.27 (0.75-2.14) |
| Model 3: Model 2+ physical inactivity, cigarette smoking, alcohol consumption | 1.58 (1.15-2.18) | 5.07 (2.44-10.56) | 1.85 (1.38-2.48) | 1.46(0.81-2.65) | 1.06 (0.14-7.96) | 1.42 (0.80-2.51) |
| Model 4: model 3 + low income level, low education level | 1.96 (1.24-3.10) | 3.58 (1.22-10.45) | 2.13 (1.39-3.25) | 1.46 (0.62-3.43) | 2.27 (0.28-18.49) | 1.53 (0.69-3.39) |

Supplementary Table 4. Association between hypothyroidism and renal function impairment/proteinuria stratified on age, sex, and glycemic status

|  | *eGFR<60* | | | *Proteinuria* ≥ *1+* | | |
| --- | --- | --- | --- | --- | --- | --- |
| *Model* | *Subclinical hypothyroidism* | *Overt hypothyroidism* | *Total hypothyroidism* | *Subclinical hypothyroidism* | *Overt hypothyroidism* | *Total hypothyroidism* |
|  | ***Men*** | | | | | |
| Crude OR (euthyroidism as reference) | 1.90(1.35-2.68) | 8.02(3.75-17.16) | 2.27(1.67-3.09) | 1.71(0.85-3.48) | NA | 1.58(0.78-3.19) |
| Model 1: age and sex | 1.16(0.78-1.73) | 5.32(2.22-12.77) | 1.44(1.01-2.07) | 1.36(0.67-2.78) | NA | 1.23(0.61-2.51) |
| Model 2: Model 1+ mean blood pressure, fasting glucose, BMI, total cholesterol, triglycerides, uric acids | 1.12(0.74-1.70) | 4.31(1.73-10.77) | 1.37(0.94-1.99) | 1.22(0.59-2.53) | NA | 1.06(0.51-2.20) |
| Model 3: Model 2+ physical inactivity, cigarette smoking, alcohol consumption | 1.13(0.73-1.76) | 3.51(1.28-9.63) | 1.33(0.89-1.98) | 1.46(0.70-3.05) | NA | 1.26(0.61-2.62) |
| Model 4: model 3 + low income level, low education level | 1.13(0.58-2.19) | 2.80(0.66-11.84) | 1.30(0.71-2.37) | 1.22(0.37-4.04) | NA | 1.10(0.33-3.60) |
|  | ***Women*** | | | | | |
| Crude OR (euthyroidism as reference) | 2.49(1.93-3.21) | 8.97(5.24-15.36) | 2.93(2.33-3.69) | 1.76(1.08-2.87) | 3.53(1.11-11.26) | 1.90(1.21-2.99) |
| Model 1: age and sex | 1.78(1.34-2.38) | 5.90(3.07-11.34) | 2.09(1.61-2.72) | 1.42(0.87-2.32) | 2.42(0.75-7.80) | 1.51(0.96-2.39) |
| Model 2: Model 1+ mean blood pressure, fasting glucose, BMI, total cholesterol, triglycerides, uric acids | 1.77(1.32-2.38) | 5.04(2.50-10.16) | 2.03(1.55-2.67) | 1.12(0.66-1.90) | 1.77(0.51-6.19) | 1.19(0.73-1.94) |
| Model 3: Model 2+ physical inactivity, cigarette smoking, alcohol consumption | 1.72(1.23-2.40) | 3.50(1.57-7.81) | 1.89(1.39-2.58) | 1.06(0.58-1.96) | 1.29(0.28-5.96) | 1.09(0.62-1.93) |
| Model 4: model 3 + low income level, low education level | 2.25(1.41-3.57) | 3.38(1.12-10.17) | 2.38(1.55-3.66) | 1.37(0.63-2.98) | 3.48(0.75-16.04) | 1.58(0.79-3.17) |
|  | ***Age: 20~49*** | | | | | |
| Crude OR (euthyroidism as reference) | 2.85(1.63-4.98) | 7.27(1.75-30.21) | 3.10(1.84-5.22) | 1.84(0.95-3.58) | 3.33(0.46-24.30) | 1.93(1.03-3.63) |
| Model 1: age and sex | 2.59(1.47-4.57) | 6.79(1.56-29.61) | 2.82(1.66-4.79) | 1.85(0.95-3.61) | 3.38(0.46-24.62) | 1.94(1.03-3.66) |
| Model 2: Model 1+ mean blood pressure, fasting glucose, BMI, total cholesterol, triglycerides, uric acids | 2.44(1.36-4.36) | 8.62(1.93-38.45) | 2.69(1.56-4.65) | 1.16(0.55-2.41) | 2.49(0.32-19.12) | 1.23(0.61-2.46) |
| Model 3: Model 2+ physical inactivity, cigarette smoking, alcohol consumption | 2.00(1.00-4.00) | 11.44(2.45-53.55) | 2.36(1.25-4.44) | 1.17(0.53-2.56) | 3.07(0.39-24.31) | 1.27(0.61-2.65) |
| Model 4: model 3 + low income level, low education level | 2.11(0.73-6.06) | NA | 1.98(0.69-5.68) | 0.82(0.19-3.48) | 8.41(1.04-68.34) | 1.19(0.36-3.90) |
|  | ***Age ≥50*** | | | | | |
| Crude OR (euthyroidism as reference) | 1.35(1.07-1.70) | 4.33(2.60-7.23) | 1.59(1.29-1.95) | 1.26(0.76-2.09) | 1.26(0.31-5.15) | 1.26(0.78-2.03) |
| Model 1: age and sex | 1.40(1.09-1.79) | 5.30(3.06-9.16) | 1.70(1.35-2.12) | 1.23(0.74-2.04) | 1.23(0.30-5.05) | 1.23(0.76-1.98) |
| Model 2: Model 1+ mean blood pressure, fasting glucose, BMI, total cholesterol, triglycerides, uric acids | 1.39(1.07-1.80) | 4.46(2.49-7.99) | 1.65(1.30-2.08) | 1.11(0.65-1.88) | 0.80(0.18-3.62) | 1.07(0.65-1.76) |
| Model 3: Model 2+ physical inactivity, cigarette smoking, alcohol consumption | 1.39(1.05-1.85) | 3.07(1.60-5.88) | 1.56(1.21-2.02) | 1.18(0.66-2.12) | 0.41(0.05-3.33) | 1.06(0.60-1.87) |
| Model 4: model 3 + low income level, low education level | 1.67(1.12-2.50) | 3.29(1.35-8.05) | 1.86(1.29-2.68) | 1.55(0.74-3.25) | 1.08(0.14-8.48) | 1.48(0.74-2.99) |
|  | ***Euglycemia*** | | | | | |
| Crude OR (euthyroidism as reference) | 2.01(1.50-2.68) | 9.11(5.18-16.04) | 2.47(1.91-3.20) | 1.69(0.90-3.19) | 3.97(0.97-16.27) | 1.87(1.05-3.34) |
| Model 1: age and sex | 1.51(1.09-2.09) | 5.89(3.00-11.59) | 1.86(1.39-2.49) | 1.45(0.77-2.74) | 2.85(0.69-11.74) | 1.58(0.88-2.82) |
| Model 2: Model 1+ mean blood pressure, fasting glucose, BMI, total cholesterol, triglycerides, uric acids | 1.38(0.98-1.93) | 5.71(2.78-11.70) | 1.71(1.27-2.31) | 1.17(0.59-2.28) | 1.88(0.43-8.15) | 1.25(0.68-2.31) |
| Model 3: Model 2+ physical inactivity, cigarette smoking, alcohol consumption | 1.43(0.99-2.07) | 5.22(2.30-11.86) | 1.72(1.24-2.40) | 1.40(0.71-2.75) | 2.20(0.49-9.83) | 1.49(0.80-2.77) |
| Model 4: model 3 +low income level, low education level | 1.79(1.08-2.97) | NA | 2.11(1.33-3.34) | 0.64(0.16-2.61) | 4.70(0.91-24.32) | 1.09(0.39-3.02) |
|  | ***Dysglycemia*** | | | | | |
| Crude OR (euthyroidism as reference) | 1.94(1.45-2.60) | 5.97(2.96-12.01) | 2.21(1.69-2.88) | 1.60(0.95-2.69) | 1.14(0.16-8.33) | 1.56(0.94-2.58) |
| Model 1: age and sex | 1.55(1.11-2.17) | 5.39(2.40-12.09) | 1.81(1.33-2.46) | 1.36(0.80-2.30) | 0.90(0.12-6.64) | 1.31(0.79-2.19) |
| Model 2: Model 1+ mean blood pressure, fasting glucose, BMI, total cholesterol, triglycerides, uric acids | 1.63(1.15-2.31) | 3.71(1.54-8.94) | 1.80(1.30-2.49) | 1.16(0.66-2.02) | 0.54(0.06-4.55) | 1.09(0.63-1.86) |
| Model 3: Model 2+ physical inactivity, cigarette smoking, alcohol consumption | 1.46(0.98-2.15) | 2.15(0.81-5.71) | 1.53(1.06-2.20) | 1.08(0.57-2.06) | NA | 0.92(0.48-1.76) |
| Model 4: model 3 + low income level, low education level | 1.66(0.93-2.99) | 1.43(0.36-5.71) | 1.62(0.94-2.79) | 2.00(0.93-4.31) | NA | 1.69(0.79-3.59) |
